# Proteome-wide identification of arginine methylation in colorectal cancer tissues from patients

**DOI:** 10.1186/s12953-020-00162-8

**Published:** 2020-05-19

**Authors:** Yongchul Lim, Ju Yeon Lee, Su Jin Ha, Suyeun Yu, Jung Kyong Shin, Hee Cheol Kim

**Affiliations:** 1 Department of Surgery, Samsung Medical Center, Sungkyunkwan University School of Medicine, 81, Irwon-ro, Gangnam-gu, Seoul, 135-710 South Korea; 2 Korea Basic Science Institute, Research Center for Bioconvergence Analysis, Ochang, South Korea; 3 Department of Preventive Medicine, College of Medicine, Korea University, Seoul, South Korea

**Keywords:** Colorectal cancer, Type I PRMTs, Arginine methylation, Immunoaffinity purification, Mass spectrometry

## Abstract

**Background:**

Protein arginine methylation reaction is catalyzed by protein arginine methyltransferase (PRMT) and the modification is implicated in various diseases including cancer. Currently, thousands of arginine methylation sites have been identified using high-resolution mass spectrometry-based proteomics technology. However, identification of arginine methylation using clinical samples at proteome level has not been reported yet. The objective of the present study was to identify, monomethyl-arginine (MMA) and asymmetric dimethyl-arginine (ADMA) sites in colorectal cancer (CRC) tissues at proteome level.

**Methods:**

Pooled CRC tissue samples from 10 patients with stage II and III were digested by trypsin and these digests were further processed and lyophilized. Using monomethyl- or asymmetric dimethyl arginine (MMA or ADMA, respectively) motif kits, methylarginine-containing peptides were enriched and subsequently analyzed by high-resolution LC-MS/MS. DLD1 and HCT116 colon cancer cells were treated with type I PRMTs inhibitor (MS023) alone or combined with SN-38, and the effect of the drugs on CRC cell proliferation and apoptosis was measured by water-soluble tetrazolium salt (WST-1) assay and FACS analysis, respectively.

**Results:**

In the present study, 455 MMA sites of 272 proteins and 314 ADMA sites of 155 proteins were identified from CRC tissues acquired from patients. In addition, 216 methylation sites and 75 substrates for PRMTs were newly identified. These results reveal the significant presence of MMA and ADMA sites on nucleic acid binding proteins and protein complexes involved in transcription. To investigate the effect of protein arginine methylation in CRC proliferation and apoptosis, MS023 was treated to two CRC cell lines. After 48 h treatment with various concentrations of MS023, CRC cell proliferation was significantly suppressed, with concomitant apoptosis induction. Furthermore, MS023 treatment significantly enhanced the inhibitory effect of SN-38 on CRC cell proliferation.

**Conclusion:**

This work reports the first comprehensive analysis of arginine methylation with clinical sample and suggests that type I PRMTs are potential therapeutic targets for drug discovery in CRC.

## Background

Protein arginine methylation is a frequent posttranslational modification catalyzed by protein arginine methyltransferases (PRMTs) which transfer one or two methyl groups from S-adenosylmethionine to guanidino nitrogen atoms of arginine within polypeptides. PRMTs are classified into two major groups (type I and type II) according to their end products. Both type I and II enzymes can generate monomethylarginine (MMA) as an intermediate. Type I enzymes (PRMT1, 2, 3, 4, 6, and 8) produce asymmetric *N*^*G*^*, N*^*G*^- dimethyl-arginine (ADMA), while type II enzymes (PRMT5 and 9) generate symmetric *N*^*G*^*, N′*^*G*^-dimethyl-arginine (SDMA) [1–3].

Arginine methylation modulates many cellular functions including transcriptional regulation, RNA processing, DNA repair, and signal transduction [1, 3]. Moreover, recent studies have implicated protein arginine modification in the pathogenesis of various human diseases including cancer [4, 5], raising the possibility that abnormally methylated proteins can be disease markers and that PRMTs might be potential therapeutic targets [6]. Consequently, identifying arginine-methylated proteins and locations of methylated residues within these proteins became more important. For proteome-wide identification of arginine methylation, mass spectrometry (MS)-based proteomics technology has been broadly applied [7]. To increase confidence in the identification of methylation sites and their relative quantification, stable isotope labeling with amino acids in cell culture (SILAC) has been frequently used. However, SILAC method cannot be applied to metabolically inactive samples such as clinical tissues from patients. Recently, the development of highly specific antibodies against methyl-arginine has made it possible to enrich arginine-methylated peptides [7, 8]. Using immune-enrichment of arginine-methylated peptides combined with MS-based proteomics technology, thousands of arginine-methylated sites have been identified from various biological sources [8–11]. Furthermore, this technology can be available for site-specific quantitative characterization of methylarginines between control and experimental groups [12, 13]. As new enrichment strategy for arginine/lysine-methylated peptides has been developed [14], it is expected that high resolution MS technique combined with efficient enrichement strategies of arginine-methylated pepties become more valuable for both basic science and biomedical research. Currently, however, proteome-wide identification of arginine methylation using clinical samples has not been reported yet.

Colorectal cancer (CRC) is one of the most commonly diagnosed cancers and a leading cause of cancer death worldwide [15]. Previously, overexpression of three major type I PRMTs including PRMT1 [16], PRMT4 [17, 18], and PRMT6 [19] was observed in CRC. The expression of one of splice variants of PRMT1 is significantly higher in colon cancer tissue compared with normal tissue. It is associated with clinical and histological parameters such as nodal status and stage [16]. Ou et al. [17] have reported that CARM1 is a positive modulator of WNT/β-catenin-driven transcription and neoplastic transformation in CRC cells. Observations from clinical samples showed that 75% of colorectal cancers had CARM1 overexpression [18]. In our recent study, patients with PRMT6-positive CRC by immunohistochemistry had shorter disease-free survival than those with PRMT6-negative CRC in both univariate and multivariate analyses [19]. All these studies strongly suggest that CRC tissues from patients might be suitable in vivo source for identification of arginine methylation.

In the present study, we report MMA and ADMA sites in CRC tissues from patients at proteome level. Using immunoaffinity enrichment of methylated peptides combined with LC-MS/MS methods, we were able to identify hundreds of methylation sites including numerous novel MMA and ADMA sites. These arginine methylation sites and substrates for PRMTs identified in this study were compared to those of an HCT116 colon cancer cell line [8]. Finally, we investigated the effects of type I PRMTs inhibitor alone and in combination with SN-38, an active metabolite of irinotecan, on CRC cell proliferation and apoptosis.

## Methods

### Human tissue samples and reagents

All clinical samples were obtained from the Samsung Medical Center Biobank for research purposes after the study was approved by the Institutional Research Board (approval number: 2017–05-065). Two human colon cancer cell lines, DLD-1 and HCT116, were obtained from the American Type Culture Collection (ATCC, Bethesda, MD, USA). Antibodies against PRMT6, Caspase 3, PARP, MMA and ADMA were purchased from Cell Signaling Technology (Danvers, MA, USA). Anti-PRMT1, PRMT4 and β-actin antibodies were from Millipore (Darmstadt, Germany), Abcam (Cambridge, UK), and Santa Cruz Biotechnology (Santa Cruz, CA, USA), respectively. CellVia for cell proliferation assay, type I PRMTs inhibitor (MS023) were purchased from AbFrontier (Seoul, Korea) and Selleckchem (Houston, TX, USA), respectively. SN-38, oxaliplatin, and 5-fluorouracil (5-FU) were obtained from Sigma-Aldrich (St. Louis, MO, USA).

### Cell culture

Two human colon carcinoma cell lines DLD1 and HCT116 were maintained in RPMI-1640 (GIBCO, Carlsbad, CA, USA) and McCoy’s 5A medium (GIBCO), respectively. All culture media were supplemented with 10% fetal bovine serum, and 1% penicillin/streptomycin (GIBCO). Cells were maintained at 37 °C with 5% CO_2_.

### Preparation of CRC tissue and cell extracts for Western blot

Ten frozen CRC tissues from patients were collected after surgery. These the tissue samples were immediately frozen in liquid nitrogen. For protein extraction, 50 to 100 mg of tissue in lysis buffer (20 mM HEPES, pH 8.0, 9.0 M urea, 1 mM sodium vanadate, 1 mM glycerol phosphate, 2.5 mM sodium pyrophosphate) was homogenized using a TissueLyser (Qiagen, Hilden, Germany) and sonicated with 3 bursts for 30 s each burst at 15 W. Extracts were then centrifuged at 20,000×*g* at 4 °C for 15 min and supernatants were stored at − 80 °C until use. Protein concentration in each lysate was measured using the Bradford method.

Cells were harvested in RIPA lysis buffer supplemented with protease and phosphatase inhibitors. Lysates were briefly sonicated and centrifuged at 13,000 rpm for 15 min.

### Purification of methylarginine-containing peptides from colon tissue extracts

To purify methylarginine-containing peptides, 3 mg of protein extracts obtained from each CRC tissue was combined to obtain a total of 30 mg of protein extracts. Then 15 mg of extract was used to analyze one type of arginine modification. Extracts were reduced with 4.5 mM DTT for 30 min at 55 °C and then alkylated with iodoacetamide (10 mM) for 15 min at room temperature (RT) in the dark. Samples were then diluted more than 4-fold with 20 mM HEPES (pH 8.0) to make the extract concentration to be 1 mg/ml followed by digestion with trypsin (10 μg/ml) overnight at RT. These digests were acidified with 1% trifluoroacetic acid (TFA). Peptides were then desalted and crudely purified from other cellular debris over Sep-Pak C18 columns (WAT051910, Waters, Milford, MA, USA), eluted with 40% acetonitrile in 0.1% TFA, lyophilized, and stored at − 80 °C. Purification of methylarginine-containing peptides was performed using PTMScan monomethyl- or asymmetric dimethyl arginine motif kits (Cell Signaling Technology, Danvers, MA, USA, #12235 and #13474, respectively) according to the manufacturer’s instructions.

### LC-MS/MS analysis

Methylation motif antibody-enriched peptides were dissolved in 0.125% formic acid with 5% CH3CN and separated on a 75 μm × 10 cm PicoFrit capillary reversed-phase column packed with Magic C18 AQ (100 A × 3 μM, Michrom, Auburn, CA, USA) reversed-phase resin. Replicate injections of each type of sample were run non-sequentially for each enrichment. Peptides were eluted using a 120-min linear gradient of (5–30) % acetonitrile in 0.125% formic acid delivered at 280 nl/min using an Easy nLC (Thermo Fisher Scientific). Tandem mass spectra were collected in a data-dependent manner with an LTQ-Orbitrap-Velos mass spectrometer. The LTQ-Orbitrap-Velos utilized a top-20 method, collecting MS spectra in the Orbitrap mass analyzer at a resolution of 60,000 [m/z (300 to 1500)] with an automatic gain control target of 1e6 (maximum ion time: 1000 ms) and collision-induced dissociation (CID) MS/MS spectra in ion trap with an automatic gain control target of 5e3 (maximum ion time: 150 ms). Dynamic exclusion parameters were employed with a repeat count of 1, and a repeat duration of 35 s. Ions with a charge of 1 or unassigned were excluded from MS/MS analysis. Polydimethylsiloxane lock mass (*m*/*z* 371.10123) was used as an internal calibrant for all runs. CID scans were acquired in a linear trap quadrupole (LTQ) with 35% normalized collision energy (NCE). A 2.0 Da isolation window for MS/MS fragmentation was applied.

### Data analysis

MS/MS spectra were evaluated using SEQUEST and the Core platform from Harvard University [20–22]. Files were searched against the SwissProt *Homo sapiens* FASTA database (released April 19th, 2015). A mass accuracy of +/− 5 ppm was used for precursor ions and 1.0 Da for product ions. Enzyme specificity was limited to trypsin, with at least one tryptic (K- or R-containing) terminus required per peptide and up to four mis-cleavages allowed. Cysteine carbamidomethylation was specified as a static modification. Oxidation of methionine and methylation (mono- or di-methyl) on arginine residues were allowed as variable modifications. Reversed decoy databases were included for all searches to estimate false discovery rates (FDR) and peptide spectral matches were filtered using a 2.5% FDR in the Linear Discriminant module of Core. Sites of arginine methylation were determined using Ascore algorithm [23] with a slight variation. Sites scoring > 13 were considered confidently assigned. Data are available via ProteomeXchange with identifier PXD011765.

### Western blot analysis

Equal amounts of CRC tissue or cell extracts were subjected to sodium dodecyl sulfate polyacrylamide gel electrophoresis (SDS-PAGE), and transferred to polyvinylidene fluoride membranes (Millipore, Bedford, MA, USA). These membranes were then incubated with respective primary antibodies at 4 °C overnight. Subsequently, these membranes were incubated with secondary antibodies at RT for 1 h. An Immobilon Western Chemiluminescent HRP Substrate (Millipore) was then used for detection.

### Cell proliferation assay

Metabolic activity of cells was assessed as an indirect measurement of cell viability using CellVia according to the manufacturer’s instructions. Briefly, CRC cells were seeded into 96-well plates at a density of 5 × 10^3^ cells in 100 μl medium per well and cultured in 5% humidified atmosphere at 37 °C. After 24 h incubation, MS023 alone or combined with SN-38 was treated with increasing concentrations as indicated in Fig. 4 for 2 days. Then 10 μL of WST-1 reagent was added to each well. Plates were further incubated at 37 °C for 3 h. Reduction of WST-1 to formazan was determined using an enzyme-linked immunosorbent assay (ELISA) plate reader wavelength of 450 nm.

### Apoptosis assay

Apoptosis was quantitated by flow cytometry using an annexin V-fluorescein isothiocyanate (FITC)/propidium iodide (PI) kit (BD Bioscience, San Jose, CA, USA) according to the manufacturer’s instructions. At 48 h post-treatment with a PRMTs type I inhibitor (MS023), cells were washed with phosphate buffered saline (PBS), suspended in annexin V binding buffer and annexin V-FITC solution, and added with PI. These cells were then incubated at RT for 15 min. Stained cells were analyzed using a fluorescence-activated cell sorter (FACS) (BD FACSVesrse™; BD Biosciences). Data were analyzed using FACSDiva™ software (BD Biosciences).

## Results and discussion

### Expression of type I PRMTs and arginine methylation profiles in CRC cell lines and tissues from patients

**Fig. 1:**
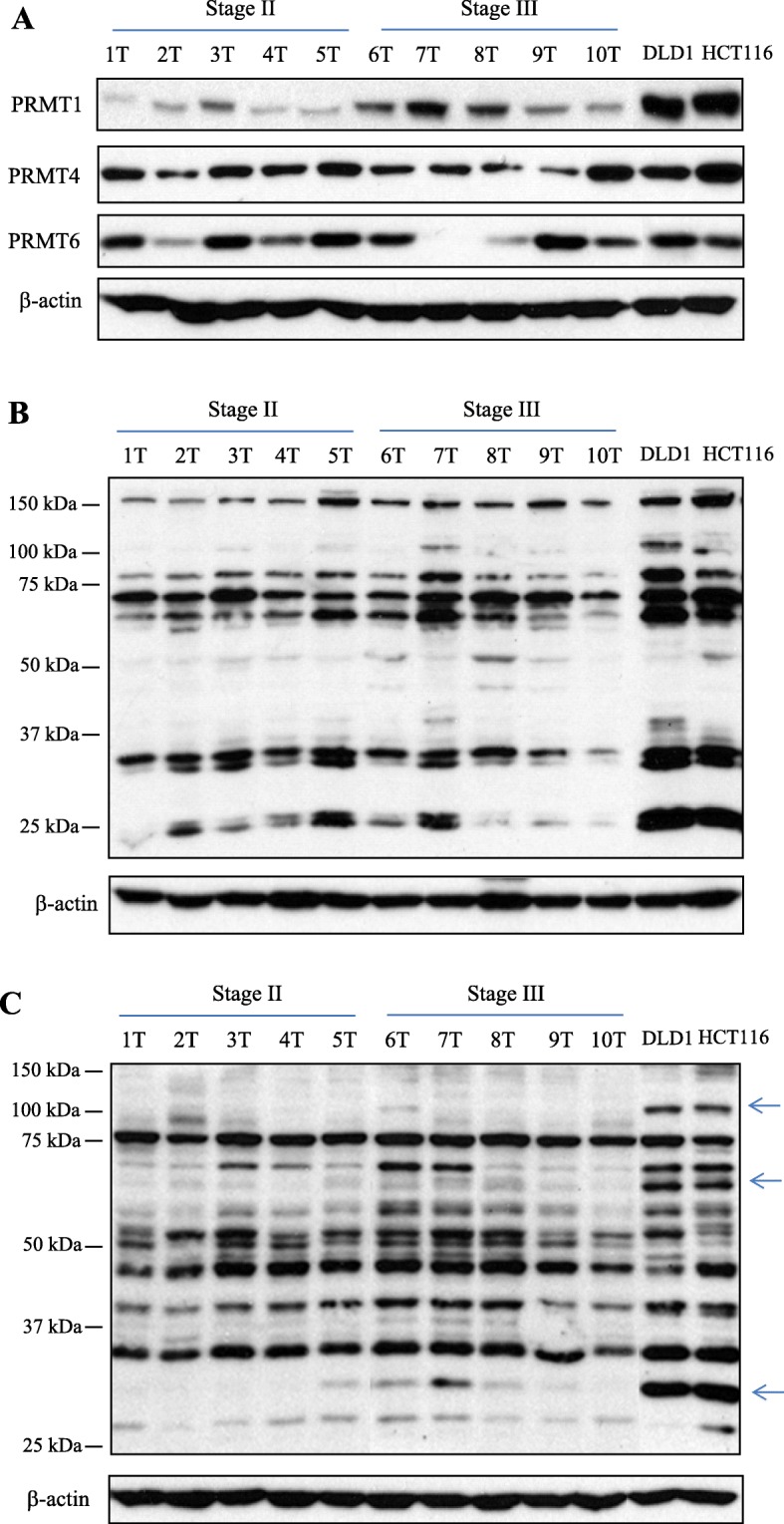
Expression of type I PRMTs, MMA- and ADMA-containing proteins in CRC tissues from patients as well as established CRC cell lines. **a** Equal amount of each CRC tissue extract from 10 patients and DLD1 and HCT116 cell lysates were subjected to Western blot analysis with the respective type I PRMT antibodies. **b** Arginine monomethylation status was examined with MMA-specific antibody. The product was conjugated to protein A agarose beads in PTMScan MMA motif kit. **c** Asymmetric arginine dimethylation status was examined with ADMA-specific antibody. The product was also used for IAP in a PTMScan ADMA motif kit. Arrows indicates highly expressed ADMA-containing proteins in two CRC cell lines, compared to those of 10 CRC tissues

Based on the previous studies [16, 18, 19], we first examined the presence of three type I PRMTs, namely PRMT1, PRMT4 and PRMT6, by Western immunoblot using CRC tissue extracts from patients with stage II and III as well as DLD1 and HCT116 cell lysates. As shown in Fig. 1a, expression level of each type I enzyme showed different expression profiles among 10 CRC tissues. In some CRC tissues, PRMT4 and PRMT6 expressions were comparable to those of two CRC cell lines. However, PRMT1 expression in two CRC cell lines was highly elevated, compared to that of CRC tissues from patients.

Next, we determined the endogenous level of MMA- and ADMA-containing proteins in 10 CRC tumors as well as for two CRC cell lines. As shown in Fig. 1b and c, arginine-methylated proteins in all the samples tested were appeared between 25 and 150 kDa molecular mass ranges. Profiles of MMA- and ADMA-containing proteins showed different patterns among 10 CRC samples (Fig. 1b and c, respectively). Comparing the immunoblot signals of arginine-methylated proteins, global levels of arginine monomethylation in CRC cell extracts was greater than those of monomethylation in CRC tissues (Fig. 1b). In addition, three ADMA-containing proteins from CRC cell extracts were highly methylated relative to those of CRC tissues (Fig. 1c). These results indicate that CRC tissues from patients and established CRC cell lines maintain different level of endogenous substrates for type I PRMTs or individual PRMTs between two biological sources may have different preferences of their substrate.

#### Identification of arginine methylation sites in CRC tissues from patients

Using MMA and ADMA motif kits which contained each methyl-arginine specific antibody conjugated to protein A agarose beads and subsequent high-resolution mass spectrometry, we aimed to identify PRMT substrates and arginine methylation sites in clinical samples by pooling 10 CRC tissues as shown in Fig. 1. Table 1 lists the number of arginine methylation sites and substrates for PRMTs identified in CRC tissue extracts. Detailed peptide information and a link to the corresponding MS/MS spectrum for all identified methyl peptides are provided in Additional file 1 (Table S1) and Additional file 2 (Table S2) for MMA, and ADMA, respectively.

**Table 1:** Arginine methylation sites identified from IAP-LC-MS/MS experiments in CRC tissues

Source	Methylation type	Peptides	New substrates / total proteins	New sites / total sites	Common proteins	Common sites
CRC tissues from patients	MMA^a^	759	462/272	122/455	68	57
	ADMA	522	29/155	94/314		

^a^Indicates total number of peptides, proteins, and sites pulled down by MMA-specific and ADMA-specific antibodies (See text). Newly identified arginine methylation sites and substrates for PRMTs in CRC tissues were searched using PhosphoSitePlus® database [24]

From the present experiment, a total of 455 MMA sites of 759 methylated peptides in 272 proteins were identified (Table 1). Many MMA sites were also identified in peptides recognized by ADMA-specific antibodies. This phenomenon can be explained by two possibilities. One is the causal presence of MMA within ADMA-containing peptides precipitated by ADMA antibodies. In this case, a peptide recognized by ADMA-specific antibodies contained both MMA and ADMA sites at different arginine residues (Additional file 2: Table S2). Another possibility is that ADMA antibodies might have partial specificity against MMA. Subsequently, these peptides contained only MMA (Additional file 2: Table S2). However, ADMA was not found within peptides precipitated by MMA antibodies, indicating that these antibodies are highly specific. A total of 314 ADMA sites were identified from 522 methylated peptides in 155 proteins. Among the total number of arginine methylation sites and substrates for PRMTs identified in CRC tissues, 216 sites and 75 substrates for PRMTs were newly identified (Table 1 and Additional file 3: Table S3). Furthermore, dozens of common proteins and sites among two types of arginine modification were identified in this study (Table 1), suggesting that the method used here was unbiased.

#### Motif and protein class analyses of arginine methylation sites

**Fig. 2:**
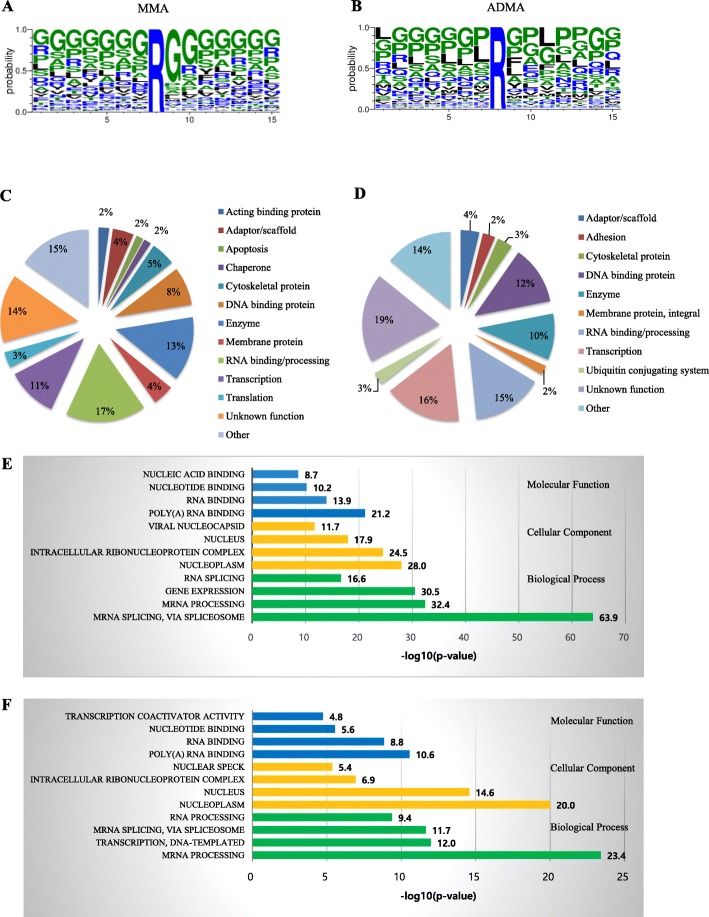
Motif and protein-type analyses of arginine methylation sites. Sequence logo for identified arginine methylation sites in CRC tissues (**a**) for MMA and (**b**) for ADMA. The 15-mer sequences were generated by Motif X. Sites that were within seven residues of protein termini were not used in the motif logo. The frequency map was generated with Weblogo. Of 455 MMA sites identified with an MMA motif kit, 54.1% had rG motif (**a**). Of 314 ADMA sites identified by an ADMA motif kit, 25.7% had the rG motif (**b**). Pie charts of protein classes for (**c**) MMA- and (**d**) ADMA-containing proteins identified in CRC tissues, respectively, based on annotations in PhosphoSitePlus® database [21]. Statistically enriched (**e**) monomethylated and (**f**) asymmetric dimethylated protein classes are categorized with Gene Ontology terms using DAVID. Significance is represented as –log (*p* value). Significance threshold is 1.301 = −log (*p =* 0.05). The length of each bar represents statistical significance. It is plotted as –log (*p* value). The number at the end of each bar shows this value

Motif analyses of peptides pulled down by MMA and ADMA specific antibodies are shown in Fig. 2a and b, respectively. The majority of MMA residues is flanked by two glycines or reside in arginine and glycine (RG) rich sequences. ADMA residues are also located in RG-rich sequences. However, dominant amino acids around ADMA are more dynamic than those around MMA. In non-RG sites found in ADMA, proline frequently occurred before and after target arginine residues (Fig. 2b).

Arginine-methylated proteins are abundant in several protein functional groups. Figure 2c and d show protein class pie charts of MMA and ADMA sites, respectively. A high proportion of arginine-methylated proteins are nucleic acid binding proteins and proteins involved in regulating transcription. A considerable number of arginine-methylated sites were also found in a variety of enzymes and proteins with unknown function. Statistically enriched protein classes of MMA- and ADMA-containing proteins were also shown in Fig. 2e and f, respectively. Enriched terms for both MMA and ADMA are RNA binding, processing, and splicing. In addition, gene expression and transcription are also represented in MMA and ADMA proteins, respectively. Consistent with these results, it is evident that methylarginine-containing proteins preferentially reside in nucleoplasm and nucleus (Fig. 2e and f).

In CRC initiation, deregulation of the WNT/β-catenin pathway is a key event and the pathway is the most studied in CRC [25]. Among the proteins identified as PRMTs substrates in this study, nuclear receptor coactivators including NCOA1, NCOA2, NCOA3, NCOA6, CREBBP and p300 are known to be linked to the WNT/β-catenin signaling cascade [26–30]. Their aberrant expressions in CRC patients and their role in intestinal physiopathology have been reported [31]. In addition, Bikkavilli et al. reported that arginine methylations of G3BP1 and G3BP2 which are also identified as PRMT substrates in this study regulate Wnt/β-catenin signaling by Wnt3a stimulation in F9 teratocarcinoma stem cells [32, 33]. Therefore, it is interesting to identify the functional importance of arginine modification in G3BPs and transcription co-regulators mentioned above during colorectal tumorigenesis via WNT/β-catenin.

#### Comparison of arginine methylation between CRC tissues and HCT116 cells

**Fig. 3:**
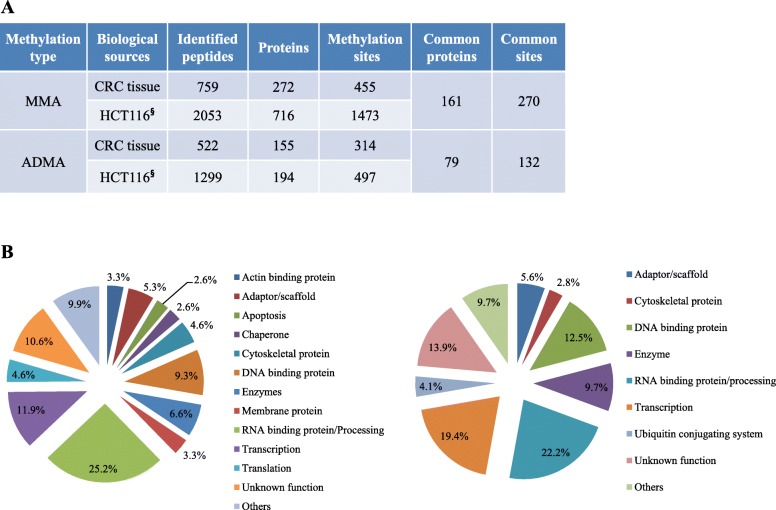
Overlap between PRMTs substrates and arginine methylation sites in CRC tissues and HCT116 cells. **a** Numbers of peptides, proteins, and arginine methylation sites identified in CRC tissues and HCT116 cells are listed. ^§^ donates HCT116 data adopted from Ref. [8]. **b** Pie charts of protein classes for MMA- (left chart) and ADMA (right chart)-containing proteins commonly identified in CRC tissues and HCT116 cells

As mentioned above, MMA and ADMA sites have been identified in HCT116 cells derived from colon cancer [8]. We compared arginine methylation sites and substrates obtained from the present study with those obtained from the previous study of HCT116 cells. As shown in Fig. 3a, the total number of methylarginine-containing proteins and methylation sites identified from HCT116 cells was higher than that of proteins and sites identified from CRC tissues (910 vs. 427 and 1970 vs. 769, respectively). This phenomenon can be partially explained by higher expression of PRMT1 and its substrates in HCT116 cells than CRC tissues (Fig. 1a and c, respectively). Previously, Pawlak et al. [34] showed that PRMT1 accounts for over 85% of the total arginine methyltransferase activity in mouse embryonic stem cells, as assayed using a synthetic substrate. Tang et al. [35] also reported that PRMT1 is the primary type I enzyme activity in mammalian cell and tissue extracts, accounting for about 90% of global ADMA deposition. On the other hand, experimental conditions including amount of antibodies used and number of IAP-LC-MS/MS repetitions might cause the differences. Nevertheless, as mentioned above, it cannot be excluded that the differences might be due to PRMT preference for its substrates and/or different level of PRMT substrates between in vivo and in vitro culture model.

We listed common arginine methylation sites and substrates for PRMTs between CRC tissues and HCT116 cells (Additional file 4: Table S4). Numbers of common MMA- and ADMA-containing proteins between two biological samples were 161 (19.5%) and 79 (29.3%), respectively (Fig. 3a and Additional file 5: Figure S1). Numbers of common MMA and ADMA sites among the two samples were 270 (16.3%) and 132 (19.4%) sites, respectively (Fig. 3a and Additional file 6: Figure S2). The majority of PRMTs substrates containing common methylation sites were nucleic acids binding proteins and proteins involved in transcription (Fig. 3b), which are similar with CRC tissue results (Fig. 2b). These findings indicate that these proteins are major targets for type I PRMTs in both clinical sample and in vitro culture model.

#### Treatment with type I PRMTs inhibitor suppresses CRC cell proliferation and facilitates apoptosis

**Fig. 4:**
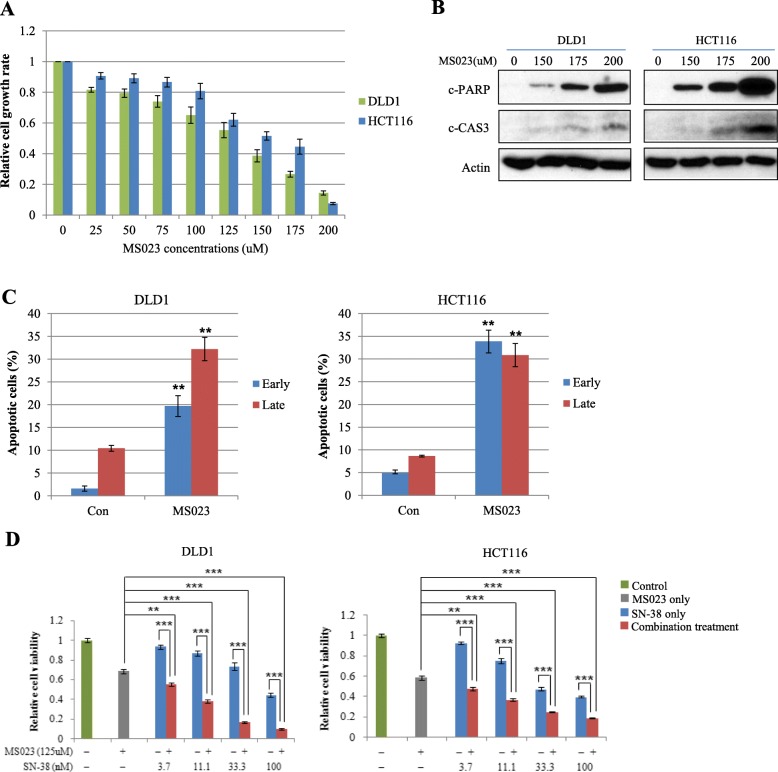
Treatment of type I PRMT inhibitor on CRC cells significantly increases CRC cell apoptosis and suppress CRC cell proliferation by SN-38. **a** DLD1 and HCT116 cells were seeded at a density of 5000 cells/well in 96 well plates. After 24 h, cells were treated with increasing concentrations of MS023 as indicated and cell viability was determined by WST-1 based assay. **b** To examine the induction of CRC cell apoptosis by MS023, two CRC cell lines were seeded at a density of 3 × 10^5^ cells/well in 6well plates. After 24 h, cells were treated with increasing concentrations of MS023 as indicated for 48 h. Induction of cleaved forms of caspase 3 and PARP proteins was analyzed by Western blot. **c** With the same condition shown in panel (**b**), mean annexin-V and propidium iodide fluorescence intensities in early and late apoptotic cell populations were quantified in three independent experiments. Representative scatter plots of each cell line are presented in Additional file 8: Figure S4. ^**^, *p* < 0.01. **d** DLD1 and HCT116 cells were exposed to increasing concentrations of SN-38 with a fixed MS023 concentration as indicated above for 48 h. Cell viability was measured by WST-1 assay. Error bars represent the mean ± SD of three independent experiments. The Student’s *t*-test was performed between cells exposed to MS023 or SN-38 alone and cells exposed to MS023 plus each concentration of SN-38 in two CRC cell lines. ^**^, *p* < 0.01; ^***^, *p* < 0.001

Significant amounts of evidence have shown that altered PRMT expression and activity are associated with tumorigenesis and consequently, PRMTs are becoming promising molecular targets in the search for new chemotherapies [6]. Eram et al. [36] have successfully synthesized a type I PRMTs inhibitor (MS023) and found that MS023 can inhibit type I PRMTs, although it is completely inactive against type II PRMTs, protein lysine methyltransferases, and DNA methyltransferases. Previously, overexpression of major type I PRMTs including PRMT1, PRMT4, and PRMT6 in CRC has been reported [16–19]. However, the effect of global down-regulation of type I arginine methylation on CRC cell proliferation and apoptosis has not been examined. To test the effect of type I arginine modification on CRC cell proliferation, DLD1 and HCT116 cells were treated with increasing concentrations of MS023. As shown in Fig. 4a, MS023 treatment exhibited inhibitory effects on the proliferation of two CRC cell lines in a dose-dependent manner (Fig. 4a). We also investigated the effect of MS023 on CRC cell apoptosis. When DLD1 and HCT116 cells were treated with three increasing concentrations of MS023 for 48 h, levels of active form of caspase 3 and PARP degradation as representative apoptosis-related proteins were clearly increased in a concentration-dependent manner (Fig. 4b), concomitant with global down-regulation of ADMA methylation in proteins (Additional file 7: Figure S3). Consistently, inhibition of type I PRMTs activities resulted in significant induction of early and late apoptotic cells in two CRC cells (Fig. 4c). These findings demonstrate that overexpression of type I PRMTs and maintenance of arginine methylation in their substrates play a vital role in CRC cell proliferation and suppression of apoptosis in CRC cells.

Next, we evaluated the combination effect of MS023 with SN-38 on CRC cell proliferation. SN-38 (7-ethyl-10-hydroxycamptothecin), an active metabolite of irinotecan hydrochloride (CPT-11), shows about 1000 times more potent than CPT-11 at inhibiting topoisomease I in vitro [37]. We used a fixed concentration of MS023 (125 μM) which resulted in about 40% reduction of CRC cell growth (Fig. 4a), and CRC cell growth inhibitory effects of MS023 were evaluated with increasing concentrations of SN-38. As shown in Fig. 4d, MS023 significantly enhanced SN-38 cytotoxicity in DLD1 and HCT116 cells. However, combination of MS023 with 5-FU or oxaliplatin which are also widely used agents for the treatment of patients with metastatic CRC showed no cytotoxic enhancement in two CRC cell lines (data not shown).

## Conclusion

To the best our knowledge, high-throughput identification of arginine methylation using clinical samples has not previously been reported yet. One major obstacle has been the lack of suitable methodologies. Recently, the development of highly specific antibodies against methyl-arginine in synthetic peptides has made it possible to enrich each type of arginine-methylated peptide followed by high-resolution mass spectrometry analysis. Applying these approaches, we identified a total of 769 arginine methylation sites present in 359 unique proteins of CRC tissues from patients. We also identified 268 new arginine methylation sites and 81 novel substrates for PRMTs. Our results indicate their significant presence in RNA-binding proteins and protein complexes involved in transcription. Furthermore, a potent type I PRMT inhibitor significantly enhanced sensitivity of CRC cells to chemotherapy agent SN-38. Collectively, our data extend the number of known in vivo arginine methylation sites and support that type I PRMTs may be useful candidates for the development of therapeutic targets in CRC treatment.

## Supplementary information

**Additional file 1:Table S1.** Summary of monomethyl arginine-containing peptide assignments from all mass spectrometric studies in this experiment.

**Additional file 2:Table S2.** Summary of asymmetric dimethyl arginine-containing peptide assignments from all mass spectrometric studies in this experiment.

**Additional file 3:Table S3.** New arginine methylation sites identified in the present study.

**Additional file 4:Table S4.** Common arginine methylation sites between CRC tissues and HCT116 cells

**Additional file 5:Figure S1.** Venn diagram showing overlaps of monomethylated and asymmetric dimethylated proteins between CRC tissues and HCT116 cells. Overlap between proteins identified by (A) MMA and (B) ADMA antibodies using protein extracts from CRC tissues and HCT116 cells. The Venn diagram was generated using online program VENNY (http://bioinfogp.cnb.csic.es/tools/venny/ index.html).

**Additional file 6:Figure S2.** Venn diagram showing overlaps of MMA and ADMA sites between CRC tissues and HCT116 cells. Overlap between arginine methylation sites identified by (A) MMA and (B) ADMA antibodies using protein extracts from CRC tissues and HCT116 cells.

**Additional file 7:Figure S3.** Down-regulation of asymmetric arginine methylation in MS023-treated CRC cells. DLD1 and HCT116 cells were treated with increasing concentrations of MS023 for 48 h as indicated above. Equal amounts of total protein (20 μg) were loaded to polyacrylamide gel followed by Western blot using anti-ADMA antibodies.

**Additional file 8:Figure S4.** Representative flow cytometry plots of control and MS023-treated CRC cells. DLD1 (A) and HCT116 cells (B) were treated with or without MS023 (200 μM) for 48 h. Detached and adherent cells were collected and stained with Annexin V and propidium iodide as described in Methods.

## Data Availability

Raw data files can be accessed via ProteomeXchange with the identifier PXD011765. All data generated or analysed during this study are included in this published article and its Additional files.

## Abbreviations

ADMA: Asymmetric dimethylarginine
CPT-11: Camptothecin-11
CRC: Colorectal cancer
DTT: Dithiothreitol
CREBBP: CREB-binding protein
ELISA: Enzyme-linked immunosorbent assay
FACS: Fluorescence-activated cell sorter
G3BP1 and G3BP2: Ras GTPase-activating protein-binding protein 1 and 2, respectively
HEPES: 4-(2-hydroxyethyl)-1-piperazineethanesulfonic acid
IAP-LC-MS/MS: Immunoaffinity purification liquid chromatography mass spectrometry/mass spectrometry
PARP: Poly (ADP-ribose) polymerase
PRMTs: Protein arginine methyltransferases
MMA: Monomethylarginine
NCOA1, NCOA2, and NCOA3: Nuclear receptor coactivator 1, 2, and 3, respectively
SDS-PAGE: Sodium dodecyl sulfate polyacrylamide gel electrophoresis
SILAC: Stable isotope labeling with amino acids in cell culture
WST: Water-soluble tetrazolium salt
5-FU: 5-fluorouracil
