# Supplementary figures and images for: Proteome-wide identification of arginine methylation in colorectal cancer tissues from patients
Source: Proteome Sci. 2020 May 19;18:6. doi: 10.1186/s12953-020-00162-8 (PMC7236946; doi:10.1186/s12953-020-00162-8)

## Slide 1
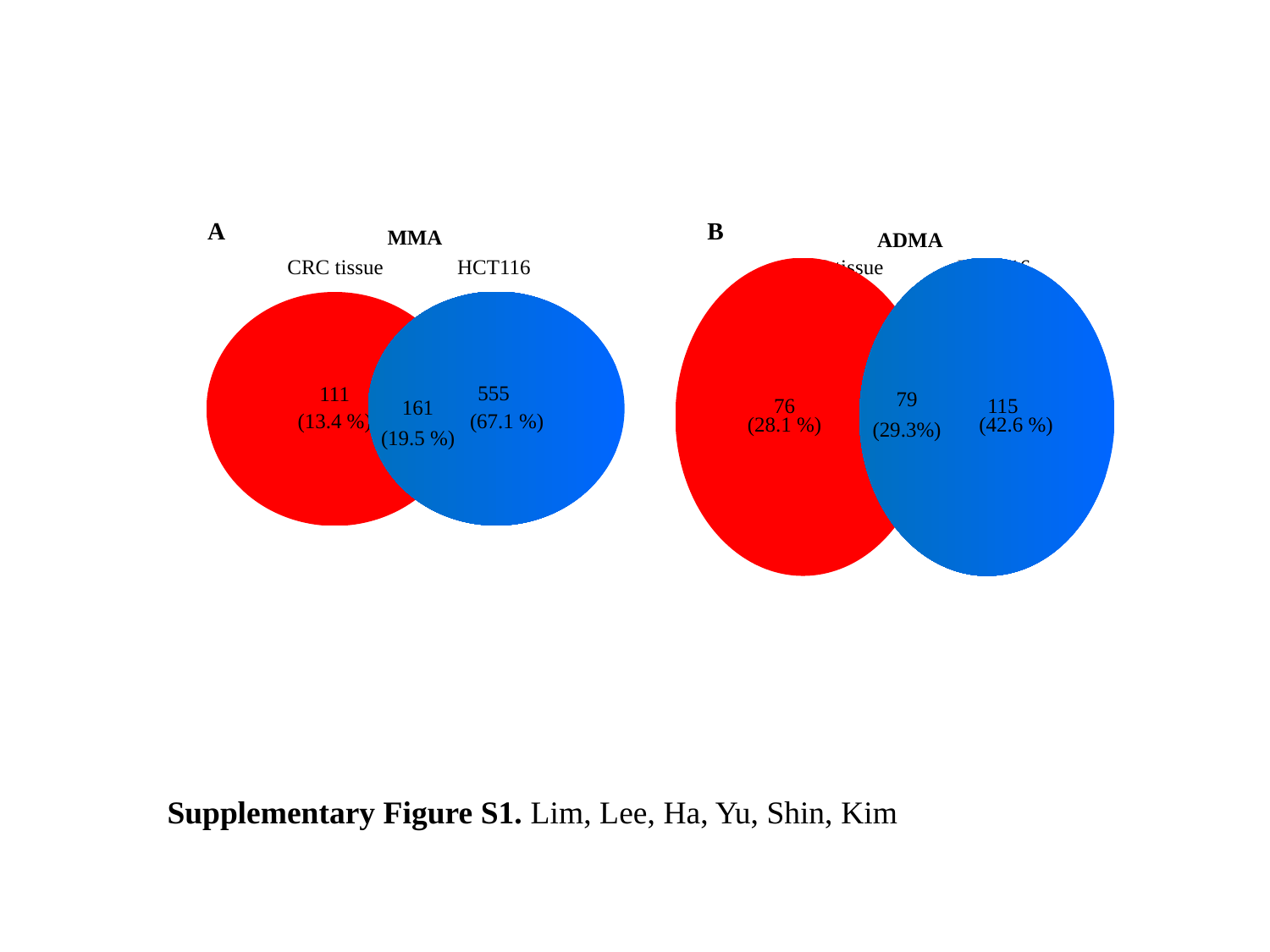

A
B
MMA
ADMA
CRC tissue
HCT116
CRC tissue
HCT116
79
(29.3%)
161
(19.5 %)
Supplementary Figure S1. Lim, Lee, Ha, Yu, Shin, Kim

Supplement: Supplementary file 5 — Additional file 5:Figure S1. Venn diagram showing overlaps of monomethylated and asymmetric dimethylated proteins between CRC tissues and HCT116 cells. Overlap between proteins identified by (A) MMA and (B) ADMA antibodies using protein extracts from CRC tissues and HCT116 cells. The Venn diagram was generated using online program VENNY (http://bioinfogp.cnb.csic.es/tools/venny/ index.html). [file 12953_2020_162_MOESM5_ESM.pptx]

## Slide 1
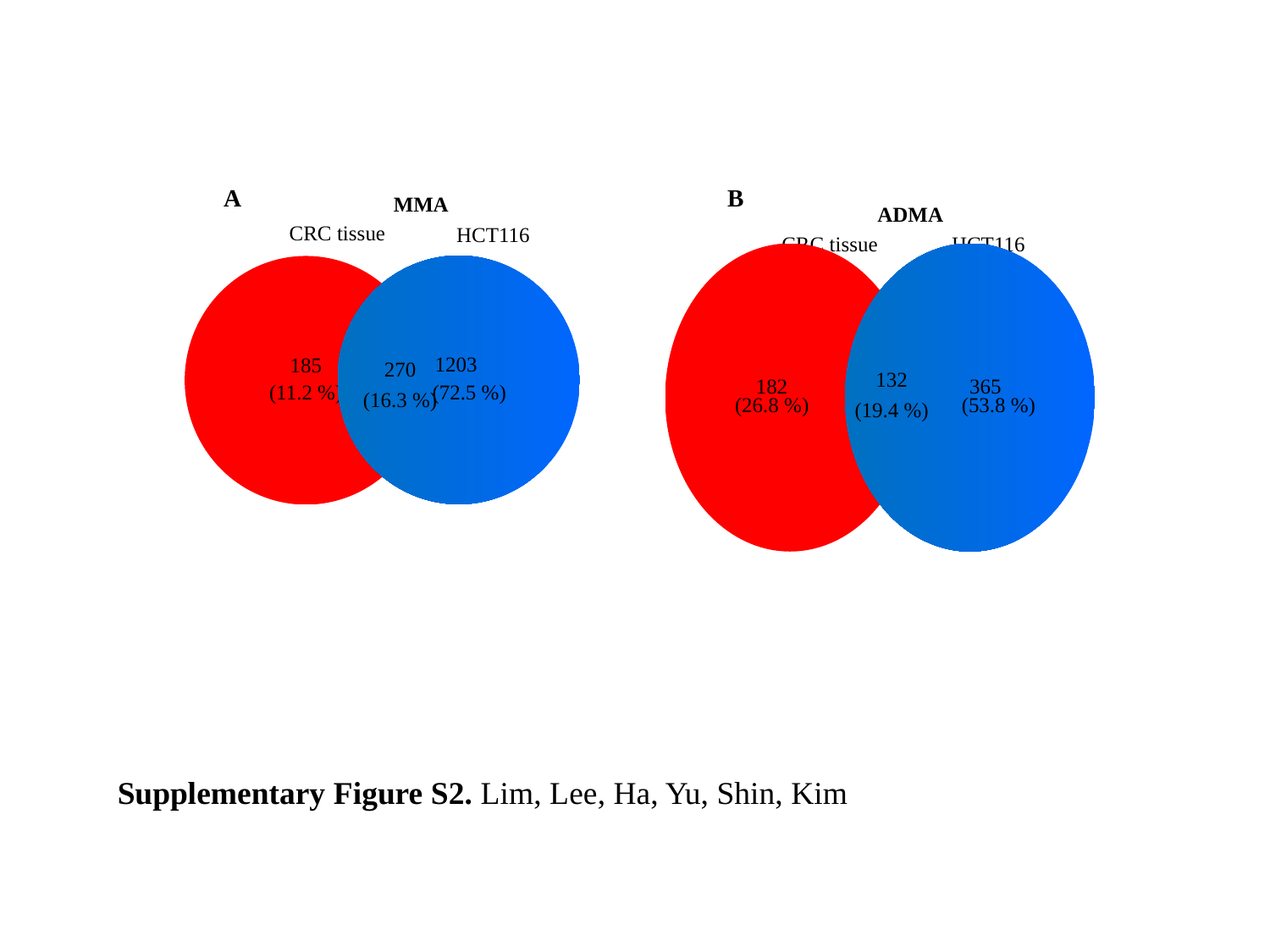

A
B
MMA
ADMA
CRC tissue
HCT116
270
(16.3 %)
CRC tissue
HCT116
132
(19.4 %)
Supplementary Figure S2. Lim, Lee, Ha, Yu, Shin, Kim

Supplement: Supplementary file 6 — Additional file 6:Figure S2. Venn diagram showing overlaps of MMA and ADMA sites between CRC tissues and HCT116 cells. Overlap between arginine methylation sites identified by (A) MMA and (B) ADMA antibodies using protein extracts from CRC tissues and HCT116 cells. [file 12953_2020_162_MOESM6_ESM.pptx]

## Slide 1
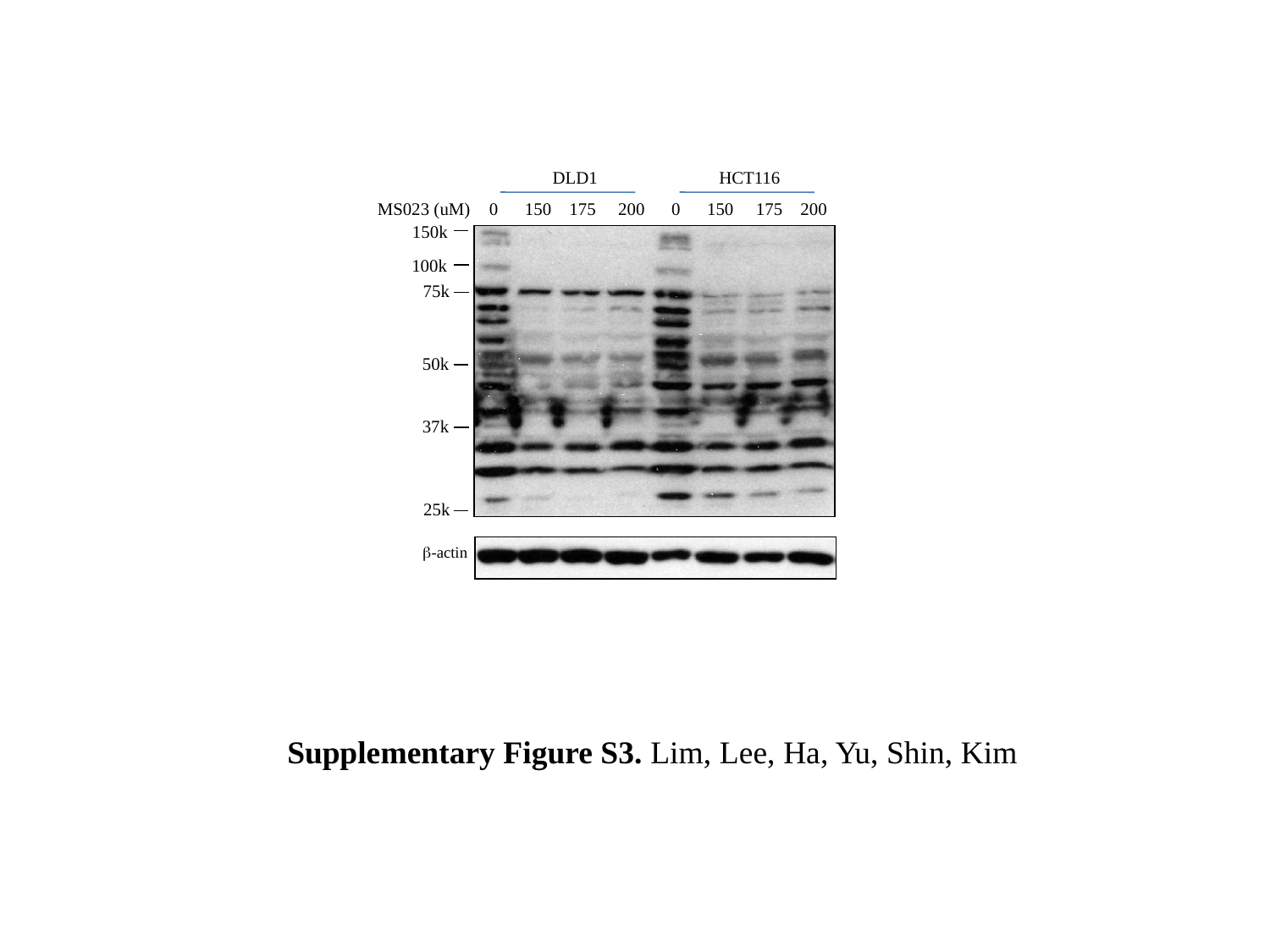

HCT116
DLD1
 0 150 175 200 0 150 175 200
MS023 (uM)
150k
100k
 75k
 50k
 37k
 25k
β-actin
Supplementary Figure S3. Lim, Lee, Ha, Yu, Shin, Kim

Supplement: Supplementary file 7 — Additional file 7:Figure S3. Down-regulation of asymmetric arginine methylation in MS023-treated CRC cells. DLD1 and HCT116 cells were treated with increasing concentrations of MS023 for 48 h as indicated above. Equal amounts of total protein (20 μg) were loaded to polyacrylamide gel followed by Western blot using anti-ADMA antibodies. [file 12953_2020_162_MOESM7_ESM.pptx]

## Slide 1
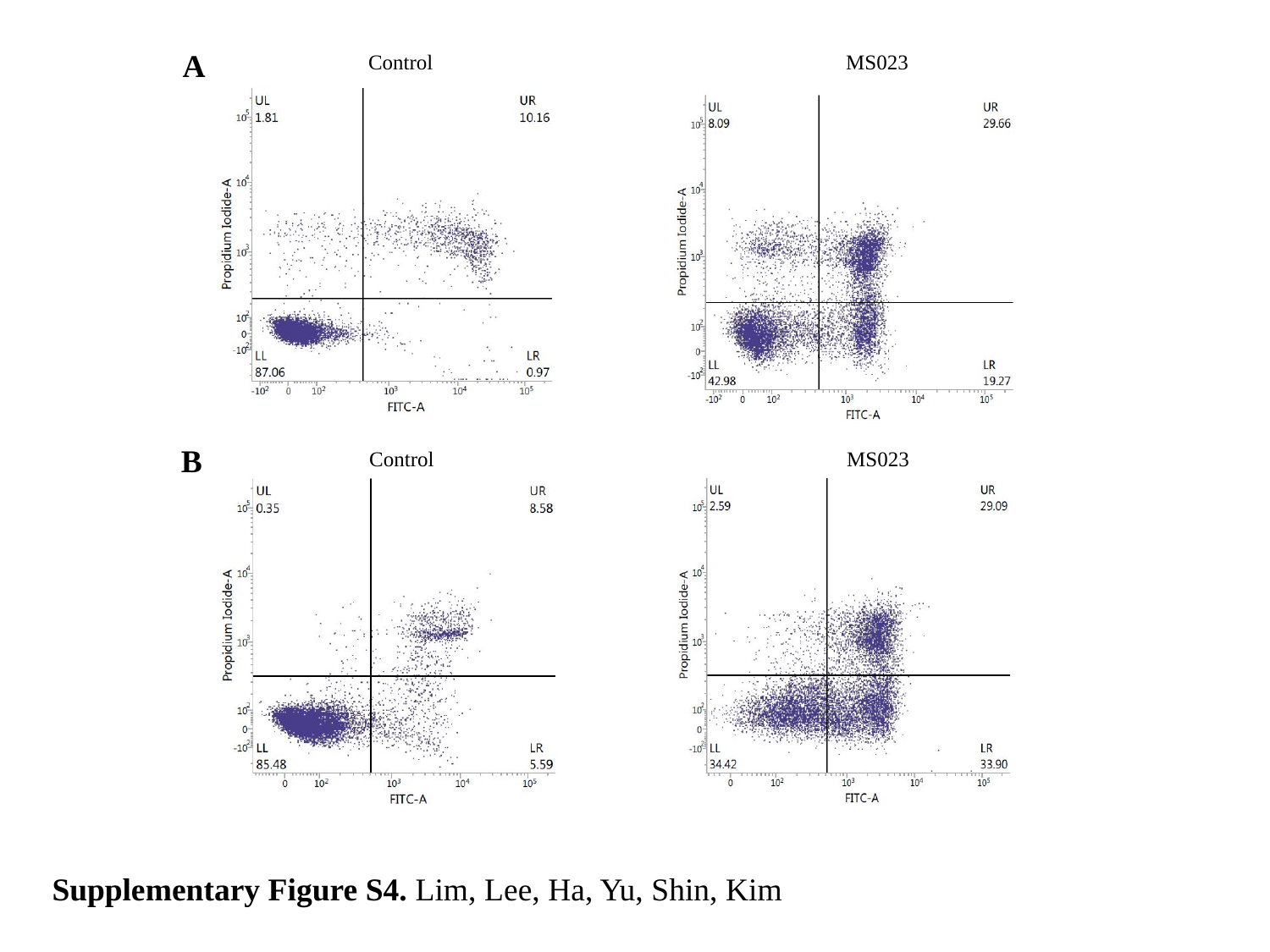

A
Control
MS023
B
Control
MS023
Supplementary Figure S4. Lim, Lee, Ha, Yu, Shin, Kim

Supplement: Supplementary file 8 — Additional file 8:Figure S4. Representative flow cytometry plots of control and MS023-treated CRC cells. DLD1 (A) and HCT116 cells (B) were treated with or without MS023 (200 μM) for 48 h. Detached and adherent cells were collected and stained with Annexin V and propidium iodide as described in Methods. [file 12953_2020_162_MOESM8_ESM.pptx]
